# A Review of Sideline Assessment Measurements for Identifying Sports-Related Concussions and Their Appropriate Management Techniques

**DOI:** 10.7759/cureus.97710

**Published:** 2025-11-24

**Authors:** Kingsley Owusu Otoo, Brandon Chou, Natalie Chou, Robert Stanley, Samuel Quaynor

**Affiliations:** 1 Department of Neurology, Georgetown University School of Medicine, Washington, D.C., USA; 2 Department of Neurology, University of Louisville School of Medicine, Louisville, USA; 3 Department of Biological Sciences, Duke University, Durham, USA; 4 Department of Biological Sciences, University of Notre Dame, Notre Dame, USA; 5 Department of Neurology, Atrium Health Floyd Medical Center, Rome, USA

**Keywords:** cognitive rehabilitation, mild traumatic brain injury (mtbi), persistent post-concussive symptoms, sideline management, sports-related concussion

## Abstract

This review responds to the evolving landscape of sports-related concussions (SRCs) by presenting a comprehensive and practical framework for sideline assessment and management. Drawing from literature published throughout 2021, our team conducted a targeted review of studies focused on the epidemiology, symptoms, diagnosis, and acute care of SRCs, to better understand their prevalence and to identify best practices for both immediate and long-term management. We synthesized the latest return-to-play and return-to-learn guidelines, particularly those outlined by the Sixth International Conference on Concussion in Sport, offering a timely and applicable reference for practitioners. A central feature of the work is a summary assessment table that organizes commonly used sideline evaluation tools, highlighting key metrics and symptoms in the domains of balance, cognition, and behavior. By simplifying and organizing critical information into accessible formats, the framework supports a wide range of users in delivering timely care and facilitating safe return to activity. Understanding the signs and symptoms of mild traumatic brain injuries is essential for ensuring accurate diagnosis, appropriate management, and improved outcomes for athletes. Ultimately, this review contributes to a more nuanced understanding of concussion recognition, evaluation, and recovery support in sports settings.

## Introduction and background

Traumatic brain injury (TBI) is a pressing health problem in the United States. An estimated 1.4 million TBIs occur each year, resulting in 1.1 million emergency department visits, 235,000 hospitalizations, and 50,000 deaths [1]. The incidence rate has risen by more than 5% within the past decade, resulting in costs of over $60 billion annually in medical and productivity expenses. TBIs are split into two categories based on the severity of the situation. Moderate to severe TBIs, which are marked by loss of consciousness (LOC) and confusion, are often the result of falls and motor vehicle crashes; about 15 cases occur per 100,000 people [2]. On the other hand, mild TBI, which is frequently associated with mild deficits and/or the lack of overt clinical symptoms, is rooted in sports and recreation activities [3]; about 131 cases occur per 100,000 people [2].

The more common of the two categories, mild traumatic brain injuries (mTBIs), are what concussions are categorized under [4]. They are a common injury suffered by athletes who participate in contact sports - football, soccer, etc. In this environment, the injury occurs from a mild blow to the head, either with or without LOC, and can lead to temporary somatic, cognitive, and emotional symptoms [5]. Depending on the severity of the impact, a variety of health consequences are associated with concussions. Short-term issues include headaches, dizziness, memory dysfunction, etc. On the other hand, long-term issues include anxiety, stress, depression, and other mental health problems [6]. If severe mental health complications persist, they may indicate the onset of a neurodegenerative disease, which can significantly reduce a person's life expectancy. One study revealed that individuals with mTBIs faced an almost 50% increased mortality risk within half a decade following the injury, primarily due to neurodegenerative diseases. It is crucial to acknowledge that an elevated mortality rate was also linked to comorbidities such as cardiovascular, neurological, and respiratory conditions [7]. 

These risks demonstrate the importance of diagnosing concussions accurately. Despite the availability of various assessment tools, inconsistencies in their use remain a significant challenge. Many existing protocols are either too complex for non-specialists or lack real-time applicability. Therefore, this updated approach is intended to guide athletic trainers, coaches, team physicians, and, ultimately, any healthcare provider responsible for immediate care decisions. By addressing gaps in existing literature and aligning with recent consensus guidelines, including the Amsterdam 2022 Consensus Statement on Concussion in Sport, this narrative review article seeks to contribute to the standardization and improvement of concussion management practices.

## Review

Epidemiology of sports-related concussions (SRCs)

Concussions are becoming more prevalent in the field of sports-related injuries. They occur at all levels and will continue to occur in even the most managed systems. Around 20% of incoming college athletes have experienced a concussion [8]. Even more worrying is the notion that many athletes typically continue regular play despite head injury symptoms. Due to youth participation in sports-related activities, the Centers for Disease Control and Prevention (CDC) approximates that about 135,000 children between the ages of 5 and 18 seek treatment in the emergency room each year to determine whether a concussion has occurred [3]. However, the number of concussions identified annually is dangerously miscalculated. Recent data argue that a more accurate estimation of about 1.6 million to 3.8 million overall SRCs occur each year, suggesting that a significant proportion of athletes remain undiagnosed [1]. 

In the United States, football, the most highly participated-in high school sport, is the leading cause of concussions among teenagers [9]. This trend even extends to collegiate athletics, where a 1988-2004 study conducted by the National Collegiate Athletic Association (NCAA) analyzed injury rates for games and practices combined for 15 sports. Over 9,000 concussions occurred, with men’s football reporting the highest number of cases at about 4,000 [10]. Select players will even go on to play at the professional level, where more concussions occur. The National Football League (NFL) has attempted to improve its concussion management through various methods, but the long-standing problem of concussions continues to exist [6]. In fact, two studies covering the years 1996-2007 recorded a total of 1,741 concussions (1,545 during games and 198 during non-games/practices) that occurred during these NFL seasons [11].

With that said, concussions, especially in the context of contact sports, are intimately related to chronic traumatic encephalopathy (CTE). CTE is a neurodegenerative disorder characterized by an excessive deposition of tau proteins in the brain. While the role concussions play in CTE is unclear - especially considering that CTE can only be definitively diagnosed postmortem - a retrospective study utilizing helmet accelerometer data from deceased football players helped shed light on the issue. Rather than the athlete's position or concussion history being directly associated with CTE status and severity, a correlation was identified between the duration of play and cumulative exposure to head impacts. This suggests that repetitive head injuries over time, as opposed to isolated concussion events, may be the mediating factor in the development of CTE [12].

Symptoms and diagnosis of concussion 

The main challenge associated with concussions, especially in a sports-related setting, is accurately determining the injury's occurrence through the available indicators and using those symptomatic cues (or lack thereof) to inform the decisions made post-injury. What makes determining whether a concussion has occurred so difficult is that the assessments depend on the brain's ability to manifest suggestive concussion symptoms [13]. The signs and symptoms that accompany concussions are often not observed immediately following a head injury. This presents the danger of incorrectly assuming that, since the symptomatology of a concussion event is absent, the injury has not occurred. Decisions following this rationale have resulted in premature return-to-play (RTP), leading to exacerbation of the unseen impairments from the initial injury and an increased risk for additional concussions. 

In addition to the diagnostic challenge, there is a widespread lack of awareness and persistent misconceptions about concussions among athletes, coaches, and even medical staff. For instance, a 2007 study found that 42% of youth sports coaches surveyed falsely believed that a LOC is required for a concussion to occur [14]. With such a prevalence of mTBI misinformation, concussion educational programs have been proposed in hopes of effectively improving injury awareness as well as patient recovery. Researchers identified that - with regard to concussion symptom awareness - headache, dizziness, nausea, vomiting, and eventual memory difficulties are generally recognized. However, there is a definitive need for improvement in educating athletes, coaches, and associated staff about the complete spectrum of signs and symptoms related to a mild head injury, and the potential consequences that may arise if it is not recognized or treated appropriately.

The development and utilization of various measures to minimize a potential injury's uncertainty and maximize the identification and assessment of concussion episodes are imperative to reduce the risk of unnecessary neurological damage due to ill-informed RTP. Over the last decade, there have been advancements in examinations that test an individual's balance, extensive neuropsychological evaluation studies, and the establishment of symptomatic consensus. Furthermore, the ability of trained sideline assessors, including physicians and athletic trainers, to effectively reduce incorrect evaluations and safeguard athletes from further harm has seen significant improvement [15]. In an analysis of the National Football League Injury Surveillance System data, researchers found that, when comparing responses to concussions during two consecutive six-year periods (1996-2001 and 2002-2007), “there was a significant decrease in the percentage of players returning to the same game, and players were held out of play longer…” [16]. This indicates increased mindfulness and precaution on the part of coaches and staff regarding the activity of players post-injury. 

Sideline assessment tools

An overview of the categories and features of symptoms is necessary to better understand how sideline assessments function. Over the last decade, several conferences have established guidelines and consensus on how to approach the assessment of a concussion event. Although each has developed its own distinct symptom categories, they all, for the most part, share the same general typology: balance impairments, cognitive dysfunction, and behavioral disturbances. For instance, according to the 2016 Berlin consensus guidelines, there are six clinical domains of concussion: symptoms of the somatic, cognitive, and emotional kind; physical signs; balance impairment; behavioral changes; cognitive impairment; and sleep disturbances [17]. The presence of one out of the six is sufficient to suspect that a concussion has occurred. The latest 2022 Amsterdam consensus continues to endorse this sentiment. Moreover, the Prague and Vienna conferences have similar views regarding the types of symptoms that are to be expected from the evolving nature of the injury. However, they describe these signs in terms of cognitive features, typical symptoms, and physical cues [18,19]. It is important to note that a comprehensive battery of assessments is available for each symptom category. For balance, one could turn to the Balance Error Scoring System (BESS) and Sensory Organization Test (SOT). For cognition, the King-Devick Test (K-D) and Standardized Assessment of Concussion (SAC) are commonly used standards. Lastly, for behavior, the Concussion Recognition Tool (CRT6) and the newer Vestibular Ocular Motor Screening (VOMS) boast high specificity in identifying mTBIs. If athletes are portraying abnormal signals in any of these areas, coaches and other sports personnel should be advised to employ at least one of the appropriate assessments outlined in Table 1 [20-23]. While none of these tests are inherently age-dependent - except BESS and SAC, which are components of both Sport Concussion Assessment Tool 6 (SCAT6) and Child SCAT6 - it is important to consider the athlete's age when interpreting them. Different age groups exhibit varying levels of physiological and cognitive development. These developmental differences, like concussion impairments, have the potential to influence an athlete's performance on these assessments. Such differences can include the possibility of less refined postural stability in younger athletes, evolving sensory integration abilities in children, and differences in cognitive processing capabilities between older and younger athletes. Conscious consideration of age in these assessments is therefore vital to avoid misdiagnosis or overlooking concussion symptoms. 

**Table 1:** Summary Assessment Table Table source: [20-23]

Category	Assessment Tool	Criteria for Suspected Concussion
Balance	BESS (Balance Error Scoring System)	If the number of errors across trials is >17. Score can be found in the modified BESS component of the Sport Concussion Assessment Tool 5 (SCAT5) assessment tool.
	SOT (Sensory Organization Test)	If the Composite Equilibrium Score is < 75, suspect concussion.
Cognitive	K-D (King-Devick Test)	If time deviation relative to baseline is >4 seconds, suspect concussion.
	SAC (Standardized Assessment of Concussion)	There are 5 assessments: Time Orientation, Immediate Memory, Concentration, Digit Backwards, and Delayed Recall. Suspect concussion if any sub-score is less than baseline, or SAC total score <26. Score can be found in the SAC component of the SCAT5 assessment tool.
Behavior	CRT5 (Concussion Recognition Tool)	Presence of red flags or observable signs
	Red Flags	Neck pain or tenderness, double vision, weakness or tingling/burning in arms or legs, severe or increasing headache, seizure or convulsion, loss of consciousness, deteriorating conscious state, vomiting, increasingly restless, agitated, or combative
	Observable Signs	Motionless, prolonged recovery after direct/indirect head contact, disorientation/confusion, blank/vacant look, difficulty with balance/movement, facial injury following head trauma
	VOMS (Vestibular Ocular Motor Screening)	If the number of symptoms present is >2, suspect concussion. Symptoms include dizziness, headaches, nausea, and fogginess.
	NPC (Near Point Convergence)	If the convergence distance is >5 cm, suspect concussion.

Many of the aforementioned assessments are integral components of widely accepted concussion assessment tools. While ongoing research aims to identify the most diagnostic measures in the areas of balance, cognition, and behavior, the consensus among researchers supports the effectiveness of evaluations in tools like SCAT6/Child SCAT6 for the initial assessment (within the first 72 hours to 1 week post-injury) and SCOAT6/Child SCOAT6 for subsequent evaluations beyond 72 hours. To operationalize these diagnostic areas, the next section describes the application and interpretation of sideline assessment tools. 

Balance Assessments

Beginning with balance impairments, this category involves irregularities in an individual’s ability to maintain their postural stability and/or issues with coordinating directed movements. Research into the exact mechanism governing balance deficits following an mTBI is currently underway. Many studies have proposed areas of the brain that may be intimately involved and thus affected during injury. Studies examining vestibular agnosia, demonstrated through the use of functional magnetic resonance imaging (fMRI) and diffusion tensor imaging (DTI), revealed that deficits in proprioception and impaired balance among acute mTBI patients are linked with altered functional connections. These alterations are observed between the bilateral lingual gyri and temporal-occipital fusiform cortex, as well as in bilateral white matter networks encompassing regions such as the right temporal lobe [24], corona radiata, thalamus, and the right inferior/superior longitudinal fasciculus [25]. While behavioral impairments, like a sudden headache or double vision, may seem to be the clearest gauges for whether a concussion has occurred, several subtle pieces of information can be gathered from measures designed for balance assessments. 

One such measure is BESS, a cost-effective and efficient determinant of postural control and stability. The test works by quantifying the number of errors made across six balance trials. These trials consist of three different positions - double-leg, single-leg, and tandem - on two distinct surface types, firm and foam, and last for 20 seconds each. Errors can range from the participant opening their eyes and lifting their hands to readjusting their position to regain balance. These errors are tallied across the trials and can be interpreted as being directly related to post-concussion postural impairment risk. In other words, a lower score is indicative of a lower risk of postural instability, and vice versa [26]. While total reliability is uncertain, the test does help differentiate between healthy and concussed athletes, and, like other examinations, reliability improves with the number of trials conducted [27]. 

The SOT is another assessment used in the evaluation of balance impairments and postural stability. SOT employs a force plate system meant to disrupt the subject’s balance and record their sensory responses. Similar to BESS, the test involves six conditions that last for 20 seconds each. The conditions vary across three eye states - open, closed, and tilted - and two surface types: tilted and flat. Subjects are scored on their sensory input, with higher scores being suggestive of proper balance and lower scores indicating impaired balance and, hence, suspicion of a concussion event. 

While both tests are useful for addressing suspicions of concussion-induced balance impairments, the advantages of BESS include its applicability due to its simplicity and integration into the SCAT6/Child SCAT6. Moreover, it requires only a piece of foam for performance [28], making it cost- and time-effective. Finally, it boasts similar reliability as the more technologically advanced SOT, especially near the time of injury. However, SOT's sophisticated framework makes it a useful supplement, particularly for extended time frames post-injury [28]. Unlike BESS, the assessment is time-consuming, requires special equipment, and is susceptible to the learning effect [29]. Moreover, the SOT may not provide a comprehensive understanding of an individual's performance in real-world scenarios, as it evaluates isolated components contributing to balance within a laboratory setting, potentially overlooking the intricate interplay of multiple sensory inputs in dynamic, everyday environments. In short, balance impairments following in-game head injuries should be assessed with BESS initially. Should symptoms persist, they can be addressed further with the SOT to avoid ruling out any balance problems missed by the BESS tool. Considering the performative attributes of each assessment, both tools excel in identifying the signs associated with acute balance deficits following a concussion. Both assessments offer objective measures beyond the more subjective evaluations, such as feelings of instability and dizziness found in CRT6, which can only be superficially assessed through immediate behavioral observation after an injury.

Cognitive Assessments

While balance assessments aptly assess the physical manifestations of concussion, cognitive assessments address the brain’s ability to process and respond to information. Cognitive dysfunction describes issues concerning mental and emotional processing, such as confusion, unawareness, amnesia, and prolonged reaction time [15,17,18]. To no surprise, there are a plethora of assessments that are reliable in relaying information regarding cognitive processing. For instance, the K-D is a two-minute examination that analyzes eye movements, language, and attention. Participants read, from left to right, a series of numbers on note cards that become increasingly challenging. The main objective is to read the note card as fast as possible with minimal mistakes. The prolonged time it takes for an athlete to get through the card helps determine whether they have suffered a concussion. Procedurally, the sideline assessment is compared to the preseason baseline to determine any significant changes resulting from the concussion. One study showed that concussed athletes performed 4.4 seconds worse on average compared to their baseline [20]. In terms of correlation, increased time corresponds to a decrease in immediate memory, which suggests that those with severe memory impairment will perform worse on the test and are believed to have suffered a concussion [30]. While the K-D test offers the advantage of quick administration and greater accuracy compared to sideline studies lacking a visual component [31], its utility in terms of result interpretation is significantly hindered without a preseason baseline. Despite being designed to assess visual function and cognitive processing, the test inadvertently relies heavily on language. Consequently, accurate interpretation becomes challenging when the test is not presented in the athlete’s native language [32]. Additionally, in cases where an athlete has a pre-existing language processing disorder, such as dyslexia, relying solely on the K-D may not adequately reflect the impact of the injury.

The SAC is another measure of the neurological effects of concussion. It is a brief assessment tailored toward detecting deficits in memory, but also addresses attention and concentration. One advantage is that it is found in the cognitive screening portion of SCAT6/Child SCAT6. The examination includes anterograde and retrograde memory exercises involving word lists and critical events, mental status activities, and postural stability inspections. The test lasts about five minutes and requires no medical expertise to perform. By first establishing a baseline score, sideline tests are employed and compared to determine the cognitive repercussions. Follow-up examinations are then used to track the athlete's recovery over time. Generally, the higher the score, the less severe the neurological damage [33]. One researcher found that a decrease in the score relative to baseline was indicative of concussion [33]. The study demonstrated 76% specificity and 95% sensitivity, suggesting that the test only missed 3 out of 63 confirmed concussion cases and invariably identified some false positives. Although the study’s specificity is high, it is outweighed by the benefits of being able to effectively identify a greater number of concussions - which is the goal of many of these assessments. The data is convincing enough to show that reducing the potential harm of premature RTP is far better than the risk of potentially picking up a few false positives. Furthermore, SAC is an essential component of the SCAT6/Child SCAT6, which is an iterative standard that has been developed to aid with the determination of a concussion.

Along with the K-D and SAC, other neurocognitive assessments have been deployed on the sidelines to determine an athlete's mental integrity following a SRC event. Examinations like the Trail Making Test, Post-Concussive Symptom Checklist, and Hopkins Verbal Learning Task are advantageous because they are designed to be quick and effective at determining whether the injury sustained is consequential to the athlete's ability to function neurologically. That is, they inform the assessor of the athlete’s ability to process crucial information necessary for physical performance [34]. Based on this assessment, researchers found concussion history and the number of symptoms to be related [34]. For example, those who have never experienced a concussion displayed fewer cognitive dysfunction markers compared with those who have experienced a concussion previously [34]. These cognitive assessments are not without their limitations. Assessors have admitted their inability to differentiate between the mild and subtle deficits a concussion may impart, and that the assessment’s reliability varies over time due to the effect of practice (retaking the assessment) on the score [35]. Nonetheless, these tests serve as indications of brain function and are often more accurate than self-reports, which tend to minimize the symptoms being experienced [35]. The risk of minimization is especially important in pediatric cases regarding acute presentation and assessment. Parents, coaches, and pediatric care providers must recognize that self-reporting symptoms of concussion episodes can be limited and unreliable; therefore, they must be assessed using the tools recommended by established guidelines. 

Behavioral Assessments

Lastly, behavioral disturbances encompass disruptions in the normal somatic or behavioral processes, and thus include everything from LOC, visual disparity, and irritability to dizziness, nausea, and even sleep disorders [15,17-19]. Although the CRT6 provides sideline assessors with a comprehensive guide, the Concussion Assessment Battery (CAB) has evolved as a critical evaluative measure for analyzing both cognitive and behavioral cues. As sideline psychological evaluations have become more accessible, more people have been assessed, resulting in an influx of valuable data. The battery is composed of three categories: self-reported symptoms, postural control evaluations, and neurocognitive assessments. What gives the battery strength in terms of sensitivity is its nine-item postural symptoms list: headache, nausea, fatigue, sleep disturbance, drowsiness, reduced reaction time, fogginess, and concentration difficulty [36]. In a study on the sensitivity of CAB, each category on its own demonstrated lower overall diagnostic effectiveness: self-report (68%), postural control (61%), neurocognitive (43.5%), compared to the collaborative battery, which boasted 89%-96% sensitivity depending on which computerized neurocognitive assessment was used [36]. So far, it has been established that no single test will accurately determine whether a concussion has occurred, and practitioners should also be aware of the limitations of the test being used, especially in cases involving younger teens and children. 

Ideally, these tests are only an additional aid in a complex diagnosis that is majorly dependent on neurological history, physical examination, and cognitive evaluation. Furthermore, behavioral assessment following a concussion is complicated by the highly individualized nature of the brain's ability to manifest symptoms. For example, when considering post-traumatic headaches, it would be incorrect to assume that the severity of a brain injury is greater simply because one athlete reports headaches, while another does not. Differences in symptom presentation may arise from individual variations in susceptibility to headaches. Consequently, researchers recommend adopting a more nuanced approach that involves identifying risk factors in a patient’s medical and injury history. Utilizing a variety of treatment modalities is also recommended to mitigate the risk of persistent symptoms [37]. With this in mind, researchers have sought to develop concussion evaluations that can help compensate for the shortcomings of those already available, and thus be used in tandem to provide a more comprehensive identification of these events. The VOMS is one of those evaluations.

VOMS examines both balance and vision, two symptoms found in most concussion cases involving adolescents and children [38]. It uses the Vestibular Ocular Reflex (VOR) and Near Point Convergence (NPC) to identify the presence of four symptoms: dizziness, headaches, nausea, and fogginess during a seven-item assessment - smooth pursuit, horizontal and vertical saccades, NPC, horizontal and vertical VOR, and visual-motor sensitivity. One advantage of VOMS is that it does not need a baseline for comparison, since it seeks to provoke symptoms suspected to be present following a concussion and screens for vestibular abnormalities not captured by BESS or the K-D test. The scores range from 0 to 10 and are based on the number of symptoms reported for each item, except NPC, which involves distance. A deviation of two points, and a distance greater than 5 cm for NPC, suggests a concussion. Higher VOMS scores tend to indicate that an individual has experienced a concussion, and often suggest prolonged recovery [39]. 

Each of the assessments described has its limitations, which can be supplemented by administering another to ensure maximal coverage of potential injury. For instance, the limitations of SCAT2 diagnostically included its need to be administered by a medical professional, the amount of time it required, its lack of an objective measure for vision and eye movement, and its inability to account for worsening of symptoms over extended periods [20]. Most of these factors can be taken into account by VOMS, which is why medical professionals have found it beneficial in clinical settings, leading to its inclusion in the latest office assessment of concussion, SCOAT6. The challenge is not only selecting the right tools, but applying them effectively under the time-sensitive conditions of the sports setting. This practical application is the focus of sideline management.

Sideline management

The rapid identification and recognition of an individual with a potential SRC is essential to the individual’s safety and the prevention of further harm. Thus, sideline management/detection is the first step in protecting athletes with a potential concussion. While any singular individual screening test or protocol cannot be definitively recommended, the best approach for a suspected concussion is to use multimodal testing under the guidance of expert agreement. The SCAT remains the most comprehensive instrument for immediate sideline assessment. However, this diagnostic measure still has its limitations. There is difficulty in differentiating between whether on-field symptoms are the result of players experiencing an intense game or players having a brain injury due to a blow to the head. Iring et al. found that increased self-reporting of red flag symptoms, such as neck pain, headaches, and muscle weakness, is present in both cases. These findings reveal the potential difficulty in appropriately identifying concussions in more acute sports settings using SCAT alone [40]. A new aspect of video review could improve sideline evaluation of head impacts and identification of concussion; nevertheless, there is a lack of evidence supporting the beneficial use of impact sensor systems for the immediate screening of concussions. Ultimately, sporting codes should collaborate to “rationalize multimodal diagnostic sideline protocols” to improve the efficiency of live sideline concussion monitoring and diagnosis [17].

When an athlete shows any signs or symptoms, or is suspected of a concussion, the first recommended step is to completely remove the player from the sporting environment. This is to be followed by first aid and emergency evaluations by a physician or another licensed healthcare provider. Once that is complete, the individual should undergo a standardized, multimodal assessment such as the SCAT6. Based on testing outcomes and clinician opinions, the treatment modalities of SRC will vary on a case-by-case basis. The sporting event should allow, and create adequate time for, proper and complete conduction of the concussion evaluation at the immediate moment when it is necessary. 

Furthermore, the necessary and sufficient facilities to adequately perform medical assessments for all injured players should be provided both on and off the field. This may cause a shift and change in rules for a few sports, to grant immediate off-field medical evaluation without affecting the remaining game or penalizing the team of the injured athlete. Such changes are important because, to an athlete, the risk of jeopardizing the team’s chances of winning may result in guilt manifesting in false self-reporting, i.e., downplaying of symptoms. Even after the completion of the immediate assessment, the individual should not be left alone, but rather, they must be closely monitored for new or worsening symptoms. Recent studies have shown a positive relationship between concussion severity and recovery time. If a player showcases on-field dizziness, visual deficits, delayed processing speed, or migraines, a longer recovery will be needed [16]. While the final diagnosis of SRC and the RTP call will be made based on clinical advising, any athlete with SRC should not RTP on the same day that the diagnosis of the concussion is made [4].

Management of concussions

While sideline management focuses on the immediate response to suspected concussions, broader concussion management strategies extend into ongoing evaluation, treatment, and recovery. Concussion management involves a variety of resources, including, but not limited to, the use of semi-regular conferences held among experts who specialize in primary care, as well as in neurological specialties. In these meetings, they explore, define, and establish standards that are used to appropriately identify, isolate, and treat concussions as they occur in a sports-related setting. Though much consensus has been reached regarding how to address concussion events, it should be noted that the manner in which different people, especially children, deal with mTBIs is highly variable and may require a different approach than the ones established in the aforementioned conferences. Nevertheless, the wealth of research that has been put into the development of sideline assessments and guidelines regarding RTP has been crucial to improving the safety and well-being of individuals who are arguably at the most risk of experiencing a concussion. While many of these tools help address the short-term symptoms, that is not to say that these signs cannot persist and develop into longer-term syndromes, some of which can invariably lead to an increased risk of other conditions, like depression. Not to mention that having a concussion history predisposes individuals to chronic executive function deficits and an increase in self-reported symptoms [31]. Moreover, some post-concussive symptoms are related to different neurobehavioral processes and even non-neurological factors. Thus, to better assess what is implicated in these symptoms, more time and data are required for these longitudinal studies [32]. Though the challenge of identifying these signs and symptoms remains, there is no doubt that the sideline evaluations following an injury, and the benchmarks collected preseason, have helped hone in on these primary concussion indicators. 

Guidelines and Consensus

To ensure these management strategies are consistent and evidence-based, international conferences have established clear guidelines and consensus recommendations. The most recent, the Sixth International Conference on Concussion in Sport in Amsterdam (2022), produced a consensus statement for physicians and healthcare providers involved in the care of athletes with SRCs. The document summarizes and compiles the current knowledge and agreed-upon procedures and management methods of SRC, as referenced in Table 2 [41]. While each SRC case should be individually assessed and treated accordingly, this consensus serves as the most up-to-date guide. The paper is organized into 12 ‘R’s of SRC management, in the logical order the steps should be considered: Recognize, Reduce, Remove, Refer, Re-evaluate, Rest, Rehabilitate, Recover, Return to Learn/Return to Sport, Reconsider, Retire, and Refine.

**Table 2:** Return to Sport/School Guidelines The graduated return-to-sport strategy is outlined by stages 1-6. The graduated return-to-school strategy is outlined by stages 1-4. Table source: [41]

Stage	Description	Focus
Stage 1: Symptom-Limited Activity	Athlete is able to perform activities that do not worsen or cause symptoms.	Gradual reintroduction of daily activities.
Stage 2: Light Aerobic Exercise	Athlete can begin walking or cycling at a slow to medium pace; No activities that require a lot of effort or resistance.	Increase heart rate.
Stage 3: Sport-Specific Exercise	Athlete can perform activities at a more rapid pace, such as running or skating; No activities that expose head to collisions.	Increase movement and range of motion.
Stage 4: Non-contact Training Drills	Athlete moves to more advanced training drills but still no head exposure; Gradually begins resistance training.	Improve physical/mental skills, balance, and coordination.
Stage 5: Full Contact Practice	If athlete is cleared by medical team, able to resume normal training activities.	Restore confidence and normalcy with supervision.
Stage 6: Return To Sport	Full return to normal play.	-

Stage	Description	Focus
Stage 1: Daily Home Activities	In intervals of 5-15 min, child is able to carry out normal activities, such as reading or texting as long as symptoms are not provoked.	Gradual reintroduction of daily activities.
Stage 2: School Activities at Home	Child can resume doing homework and exercise cognitive thinking.	Increase cognitive work tolerance.
Stage 3: Return to School Part-Time	Child can attend a partial school day or a school day with an abundance of breaks.	Gradual return to coursework and school activities.
Stage 4: Return to School Full-Time	Child can begin attending normal school days.	Return to normalcy and catch up on missed assignments.

Recent updates to the NCAA’s Concussion Safety Protocol Checklist and Protocol Template (effective January 2024) explicitly incorporate recommendations from the Amsterdam 2022 Consensus Statement, as reviewed and endorsed by the NCAA’s Concussion Safety Advisory Group [42]. This demonstrates the direct influence of international standards on domestic concussion policy and underscores the growing alignment between global and North American concussion management practices. 

Return to Sport (RTS)

Athletes who suffer from SRCs are typically recommended to consider a graduated, stepwise rehabilitation and recovery strategy, individualized and timed to their symptoms. It is generally advised that all patients undergo a period of cognitive and physical rest, typically lasting 24-48 hours, before progressing through the symptom-monitored, graduated RTS strategy. The standard procedure for RTS follows a six-stage progression, in which each step should be at least 24 hours, with constant monitoring of symptoms. Thus, the minimum time frame for a complete RTS would be a week [43]. The Sixth International Conference on Concussion in Sport of 2022, held in Amsterdam, constructed a comprehensive table that outlines the recommended graduated, stepwise RTS strategy, as summarized in Table 2 [37].

As previously mentioned, symptom monitoring throughout the progression is an important part of the athlete’s recovery. If symptoms worsen at any point during the progression, it is suggested that the individual return to the previous stage. Researchers have observed that athletes may be more likely to report their symptoms in writing than verbally to an individual [44]. Therefore, to track and ensure the absence or reduction of symptom severity, the concussed individual might be provided with a post-concussion symptom-scale survey, periodically, throughout each stage of the stepwise RTS strategy.

While each sport may choose to adjust and specialize each stage and its included activities, considering factors like technique or fitness standards specific to the sport, it is important to note that these adjustments should explicitly consider and monitor factors such as the type of activity, intensity, duration, and environmental conditions during exercise. Any adaptations or specifications from sport-specific programs should still align with the basic aims and goals of the six stages established for RTS. Moreover, it is generally recommended that final clearance for RTS be provided by a medical professional, preferably a physician or advanced-practice provider.

Pediatric considerations

While SRCs are extensively researched in college and professional athletes, the majority of players involved in contact and collision sports are adolescents and children. Understanding and addressing SRCs in the pediatric setting is, therefore, crucial. One researcher emphasizes diverse risk factors, like age, sex, sport level, athlete-specific factors, equipment, and sport type. The study notes that football, ice hockey, lacrosse, and soccer pose the highest concussion risks, with girls exhibiting a higher rate of concussions than boys in comparable sports [17]. 

Early concussion management is crucial to the patient’s path to recovery. Fortunately, current youth legislation in most states mandates immediate removal from play if a concussion is suspected, along with evaluation and clearance by a licensed healthcare provider, before returning to play. Many studies also indicate that reassurance, injury education, and psychological counseling at the beginning can reduce the risk for later development of post-concussion syndrome. Research further suggests that minimizing cognitive effort and activity corresponds to fewer symptoms and faster concussion recovery. 

Recovery, starting with “return to learn” in the academic setting, should precede “return to sport” in a gradual manner, following the resolution of acute symptoms, without symptom-masking medication. The gradual RTP protocol encourages moderation of cognitive activity and symptom-based supportive care. Ultimately, pediatric concussion management should be individualized, considering risk factors for prolonged recovery.

Persistent post-concussive symptoms (PPCS)

Beyond initial recovery, a subset of both pediatric and adult athletes experience symptoms that linger well beyond the expected timeframe. These cases are described as PPCS. PPCS are symptoms that extend beyond the expected 10-14-day resolution period for adults, and the 30-day period for children [44,45]. While some define PPCS within the first three months post-injury, the absence of a standardized definition hinders generalization in identification and treatment. Given that these persistent symptoms are unlikely to result from a single pathophysiological etiology, a comprehensive, multimodal clinical assessment - encompassing somatic, cognitive, cervical, vestibular, oculomotor, and hormonal domains - as well as a detailed history, focused physical examination, and indicated special tests, is crucial for identifying contributing factors to prolonged symptoms.

Once the necessary assessments have been performed, treatment and recovery should be targeted toward the specific pathologies identified from the tests. Studies have shown that a collaborative approach, combining symptom-limited aerobic exercise, targeted physical therapy, and cognitive-behavioral therapy, aids in the recovery of PPCS patients. Notably, evidence supporting pharmacotherapy for recovery is currently lacking. Furthermore, a study on sub-symptom-threshold exercise training for refractory PPCS found controlled aerobic exercise to be beneficial for patients with lasting physiological disequilibrium. Ultimately, managing PPCS requires a case-by-case evaluation and a collaborative effort among healthcare providers experienced in SRCs, within a multidisciplinary setting.

## Conclusions

The widespread prevalence of concussions in sports underscores the need for clear, evidence-based guidelines for assessment and management, especially as many coaches and parents remain unfamiliar with appropriate response protocols. Medically endorsed tools, such as CRT6 and SCAT6/Child SCAT6, along with immediate removal from play and continuous monitoring, are critical steps that enhance recovery outcomes and reduce long-term risk. While standardizing concussion protocols remains challenging due to the diverse profiles of athletes, the 2022 Amsterdam guidelines emphasize individualized approaches that account for factors such as age and symptom variability. Strategies like prompt reassurance, injury education, and psychological counseling have shown promise in reducing the risk of PPCS. With that said, the complex nature of PPCS demands a multidisciplinary approach, involving symptom-limited aerobic exercise, targeted physical therapy, and cognitive-behavioral therapy. This review highlights the variability and complexity of concussion cases, reinforcing the need for flexible yet informed approaches to identification, treatment, recovery, and RTP decisions. When implemented correctly, the outlined guidelines, assessment tools, and management strategies significantly improve the likelihood that any individual will be able to RTP.
